# Kinesthetic engagement in Gestalt evaluation outscores analytical ‘atomic feature’ evaluation in perceiving aging in crystallization images of agricultural products

**DOI:** 10.1371/journal.pone.0248124

**Published:** 2021-03-15

**Authors:** Paul Doesburg, Jürgen Fritz, Miriam Athmann, Roya Bornhütter, Nicolaas Busscher, Uwe Geier, Gaby Mergardt, Claudia Scherr

**Affiliations:** 1 Institute for Integrative Medicine, University of Witten/Herdecke, Herdecke, Germany; 2 Department of Organic Farming and Cropping Systems, University of Kassel, Witzenhausen, Germany; 3 Institute of Crop Science and Resource Conservation, Department of Agroecology and Organic Farming, University of Bonn, Bonn, Germany; 4 Forschungsring e.V., Brandschneise 5, Darmstadt, Germany; 5 Department of Organic Food Quality and Food Culture, University of Kassel, Witzenhausen, Germany; 6 Society for Cancer Research, Arlesheim, Switzerland; University of Wuerzburg, GERMANY

## Abstract

There is an increasing interest in a systemic approach to food quality. From this perspective, the copper chloride crystallization method is an interesting asset as it enables an estimation of a sample’s ‘resilience’ in response to controlled degradation. In previous studies, we showed that an ISO-standardized visual evaluation panel could correctly rank crystallization images of diverse agricultural products according to their degree of induced degradation. In this paper we examined the role of contextual sensitivity herein, with the aim to further improve the visual evaluation. To this end, we compared subjects’ performance in ranking tests, while primed according to two perceptional strategies (levels: analytical vs. kinesthetic engagement), according to a within-subject design. The ranking test consisted out of wheat and rocket lettuce crystallization images, exhibiting four levels of induced degradation. The perceptual strategy imbuing kinesthetic engagement improved the performance of the ranking test in both samples tested. To the best of our knowledge, this is the first report on the training and application of such a perceptual strategy in visual evaluation.

## Introduction

There is an increasing interest in a systemic approach to food quality, either from an organic food quality perspective [1–3], or from the need for a better understanding of the nutrition-health interface, e.g. via the food matrix [4–6]. Complementary to a direct rating of a food’s outer appearance (e.g. color change, signs of microbial mediated decay), the method of copper chloride crystallization with additives enables an estimation of a sample’s ‘resilience’ (elasticity, capacity to cope) in response to controlled aging of the sample [7–12].

The method is based on the evaluation of the crystallization patterns that arise when a food juice or extract is mixed with an aqueous cupric chloride solution in a Petri-dish under controlled climatic conditions [13].

To attain a scientific communicable, objective means of visual evaluation, a panel was formed and validated according to ISO-norms for sensory panels, adapted towards the visual evaluation of crystallization images [14]. This morphological level of evaluation was subsequently extended towards the perception of salient coherent ‘meaningful wholes’ or Gestalts, relating to the plant physiological processes of ripening and decomposition, as perceived in the crystallization images [7, 8].

Previously we showed that this panel could correctly rank crystallization images of different agricultural products according to the degree of controlled aging [8]. Although the results reported were encouraging, the accuracy varied considerably among the panel members.

This triggered research into the effect of contextual sensitivity in the visual evaluation. To this end, we compared subjects’ performance while primed according to two perceptional strategies (levels: analytical vs. kinesthetic engagement), according to a within-subject design. Kinesthetic engagement is an observer’s kinesthetic sensation, or motoric response, towards observed motions or implicit motions of human, non-human and even inanimate objects [15, 16]. Kinesthetic engagement in crystallization image evaluation involves an embodied simulation of the growth, curvature and tension of the tree-like branches of the crystallization images [17].

### Crystallization with additives

The method of crystallization with additives generates two-dimensional dendritic crystallization patterns when an aqueous dihydrate cupric chloride solution is mixed with a juice or extract in a Petri-dish under controlled climatic conditions [13]. The properties of the patterns are influenced by the type and properties of the additive [18, 19]. With aging of the juice or extract, the crystallization patterns change in a characteristic and reproducible manner [7–11, 20], thereby making the method an interesting asset in food quality assessment from an ontological holistic stance.

Kahl et al. [3, 21] discussed validation strategies for systemic approaches to organic food quality determination, including the method of copper chloride crystallization with additives. This stimulated an ongoing development of a European consortium of crystallization laboratories towards standardized methodology with respect to sample preparation [13, 22–26], crystallization [18, 19, 27–29], data evaluation including multivariate statistical analysis of computerized image analysis [30–33], and visual evaluation using defined morphological criteria [7, 8, 14].

Although there is still a lack of knowledge concerning the physical basis of the pattern formation process; e.g. for authenticity tests, the macroscopic appearance of the crystallization patterns depends on the amount and type of the additive and the amount of dihydrate cupric chloride [18, 19]. The additive changes the surface properties of the glass plate [20, 34], while the additive’s viscosity (molecular weight) influences the dendritic branching conditions of the crystallization process, which results in sample-specific crystallization patterns [18, 20, 29, 35, 36].

For the quantification and classification of the morphological features of the crystallization patterns, computerized analysis allows an unambiguous objective means of evaluation and the possibility to evaluate large data sets for e.g. methodological studies. The visual evaluation remains however superior in differentiating samples based on their resistance to degradation. Trained individual researchers could correctly assign encoded samples of wheat, grape juice and rocket lettuce based on the perceived degree of decomposition [9–11, 37].

### Visual evaluation

A standardized and scientifically communicable approach to the visual evaluation of crystallization images was established by Huber et al. [14] and has been comprehensively described and extended by Doesburg et al. [7] to the level of Gestalt evaluation. ISO 8587 [38] “Sensory Analysis–Methodology–Ranking” was adapted for the development of a ranking measurement instrument for Gestalt evaluation. The norm aims at placing a series of test samples in a ranked order based on the intensity of an overall impression. This was utilized for the Gestalt decomposition from fresh to decomposed.

A Gestalt is defined as ‘a perceptual pattern or structure possessing qualities as a whole that cannot be described merely as a sum of its parts’ [39]. The Gestalt laws of perceptional organization account for man’s innate ability to perceive organized patterns (Gestalts) in visual stimuli [40].

The relevant features characterizing the Gestalt decomposition in crystallization pictures are described according to four levels of evaluation criteria (Table 1), reflecting a hierarchical complexity [Doesburg et al. [7] level 1–3, this study level 4].

**Table 1:** Characteristic features of increased decomposition in wheat crystallization images.

1. Level	2. Level	3. Level	4. Level
Quantifiable single morphological and local features	Quantifiable, descriptive single morphological features	Gestures or implicit motions in the whole pattern	Kinesthetic criteria
Decrease of coarse structural features	Increase of ‘Wickerwork’	Decrease of integration	Decrease of fluent interconnected movement
Decrease of the number side needles	Increase of the angle of side needles	Decrease of ‘Perradiation’	Decrease of a sense of presence in the image
		Decrease of center coordination	Decrease of tension in the needle branches from the center to the periphery
		Decrease of ‘Organic curvature’	Decrease of a consistent dynamic in the filling of the plate

Note: Nomenclature level 1–3 according to [14] in [7], level 4 in present study.

### Two-way model of perception

It is commonly understood that the visual perception of complex images can be described by a two-way model [41, 42]: object recognition is performed via a selective pathway, mainly utilizing foveal vision (single object identification, feature guidance, analytical perception). In the second path of perception, foveal object recognition is guided by a non-selective path (shape, or "gist of a scene"), which extracts global or statistical information from the entire visual field [43]. Global, non-selective image processing allows observers to assess the mean and distribution of a variety of basic visual feature dimensions. Gestalt perception can be considered a specific form of global processing, and forms a key principle in diagnosing and solving unique, complex and context-specific problems in disciplines where a parts-alone view does not suffice [7]. The Gestalt decomposition, as reflected in crystallization images of diverse agricultural products, appears as a salient unity of multiple emergent interrelations between local criteria and global features. However, it does not dismiss the fundamental role of local features, but contrary to object recognition these features are not perceived and represented independently from each other [44].

The mode of processing, either analytical or holistic, has not been controlled in the practice of Gestalt evaluation of crystallization images so far. Moreover, it is well documented that when an individual is presented with a stimulus containing both global and local features their mode of processing is seemingly dictated by task, culture, level of expertise and personal preference [45–47].

In the present study, we compared subjects’ performance in ranking tests, while primed according to two perceptional strategies (levels: analytical vs. kinesthetic engagement), according to a within-subject design. In order to prime the perceptual strategy, the subjects were instructed to evaluate the crystallization images according to either an analytical perceptual strategy (single object recognition) by adhering to the use of ‘atomic features’ (i.e. utilizing solely the criteria of the levels 1 and 2 in Table 1), or according to a kinesthetic engagement in Gestalt perception and a subsequent secondary, confirmatory ‘atomic feature’ evaluation (i.e. mainly levels 3 and 4 in Table 1).

Contrary to single object recognition, in Gestalt perception local features are not perceived and represented independently from each other [44]. Therefore we expected to observe that a kinesthetic engagement in Gestalt perception globally would improve the degree of correct ranking.

## Materials and methods

### Origin of the wheat samples

Wheat grain samples (*Triticum aestivum* L. cv. ‘Titlis’) were obtained from a long-term field trial comparing different farming systems in Oberwil/Switzerland (the so-called DOK trial) from harvest year 2005. For a more detailed description see [9].

### Origin of the rocket lettuce samples

Rocket lettuce samples (*Eruca sativa* L.) were obtained from a field trial carried out in autumn 2009 at the Wiesengut organic experimental farm of the University of Bonn (D). More detailed descriptions can be found in [37].

### Crystallization method

Wheat grain kernels were ground using a hand grain mill with a stone mill work (Schnitzer hand grain mill type ‘Country’) with a standard adjustment. 5 g of wheat whole flour was extracted with 25 ml distilled water at 28°C for 3.5 h. The 3.5 h extracted samples were filtered with paper filters grade 604 (Schleicher & Schuell, Dassel, Germany) and subsequently stored at 8°C for 0, 3, 8 and 12 days before being used as additive in the crystallization.

The rocket lettuce leaves were either i) pressed in a juice press (Porkert, Czech Republic) and stored at 4°C for 1 day, ii) stored in plastic boxes for 4 days, pressed and subsequently stored at 4°C for 1 day, iii) pressed and stored for 6 days and iv) pressed and stored for 10 days. Leaves and juices were stored at 4°C. For detailed methodological descriptions see [9] for wheat [37], for rocket lettuce. All samples were crystallized in the crystallization laboratory at the University of Bonn in Germany. After crystallization, all images were photographed against a dark-field illuminated background (JPEG format 2268 x 1512), and printed on photographic paper for evaluation.

### Visual evaluation panel

A number of 6 (wheat ranking test), or 8 (rocket lettuce ranking tests) experienced panel members, trained in recognizing the Gestalt decomposition in crystallization images, took part in the experiment (mean age respectively 49.8 and 49.1 years, SD 8.8 and 9.1; respectively 33% and 50% female). Participants had normal or corrected-to-normal vision.

### Training and exam sets

For training and exam, prints of wheat and rocket lettuce crystallization images exhibiting increasing degrees of decomposition were collected. For wheat, crystallization pictures exhibiting 0, 3, 8, and 12 days of wheat extract aging prior to crystallization were used. For rocket lettuce, crystallization pictures exhibiting 1 day of juice aging; 4 days of leave aging and an additional 1 day of juice aging; 6 days of juice aging, and 10 days of juice aging prior to crystallization were used. From each decomposition stage, three crystallization images were presented to the visual evaluation panel, comprising three increasing additive amounts.

### Visual evaluation–Training and exam

The training was performed as described in [8], via concept-learning according to supervised classification [48, 49] by email, video conferencing and real-time meetings. Encoded training sets with decomposed samples were presented in random order to the panel, or mailed as portable document format (PDF) files. All samples of a set were taken from one of the (organic or conventional) agricultural production systems, only the intensity of the decomposition was varied. The amount of CuCl_2_.2H_2_O was the same in all images. Images of three mixing ratios of additive per sample were presented. The training sets for ranking the decomposition levels consisted of eight sets of wheat crystallization images and eight sets of rocket lettuce crystallization images. Each set contained crystallization images exhibiting varying degrees of decomposition as indicated above.

During training, the list of characterizing features (Table 1) could be used. The images were evaluated by each panel member independently, then decoded and discussed. Thereby applying concept learning by supervised classification and peer tutoring by the best performing evaluators.

Panel tests (exams) were performed for wheat on April 4 and 5 2016 (panel size n = 6); the rocket lettuce examination was performed on March 13 and 14 2018 and repeated on September 27 and 28 in the same year (panel size n = 8). Each panel test lasted two days, and was performed in single cabins of the sensory laboratory at the University of Kassel, Germany, designed according to ISO 8589 [50] “about requirements for sensory cabins”. Panel tests were performed without using the list of characterizing features. Each of the panel members simultaneously received encoded evaluation sets in random order. The wheat panel tests consisted of 10 sets. The rocket lettuce exams consisted of 20 sets. Each exam set consisted of four decomposition levels, which in turn encompassed three images for the three mixing ratios. The crystallization images used for training and panel tests were obtained from the same experimental series according to ISO-norm 11035 [38].

### General design of the study

In this paper we examined the role of contextual sensitivity in ranking tests by contrasting subjects’ performance according to an analytical and a holistic (kinesthetic engagement) perceptual strategy. The subjects went through the experimental block in a way that enabled a within-subject comparison.

### Primary outcome parameter

To determine the classification error in the ranking order, the RMSE (Root Mean Square Error) was used, calculated on the base of the subjects’ confusion matrices. A confusion matrix is a matrix in which *C*_*ij*_ represents the number of instances which are known to be in group *i* (true label) and predicted to be in group *j* (predicted label).

Compared to the more commonly used MAE (Mean Absolute Error) and the MSE (Mean Square Error) the RMSE has as the advantage that the larger classification errors are given extra weight. Calculation based on a confusion matrix has as an additional advantage that the absolute differences between true and predicted class numbers are taken into consideration, e.g. it is worse to predict points from class C1 to belong to class C3 rather than to predict them to belong to class C2.

$$\text{RMSE}:\sqrt{\;\frac{1}{N}\sum_{r=1}^{K}\sum_{c=1}^{K}n_{r,c}{\left(r-c\right)}^{2}}$$

K is the number of classes. N is the sum of the instances in the matrix. Each entry n_r,c_ represents the number of points from the r^th^ class predicted as being from c^th^ class) [51].

### Statistical analysis

Statistics were performed with Rstudio Version 1.3.1093 [52]. The mean RMSEs for the two perceptual strategies were compared by means of a paired sample t-test from the “rstatix” Rcran package version 0.7.0 (https://www.rdocumentation.org/packages/rstatix). Graphs were plotted with the RStudio packages “ggplot2” version 3.2.1 [53], “ggpubr” version 0.4.0 (https://www.rdocumentation.org/packages/ggpubr), and “cvms” version 1.2.1 (https://www.rdocumentation.org/packages/cvms/versions/0.2.0)

## Results

### Inventorying kinesthetic criteria

Kinesthetic criteria characterizing the Gestalt decomposition were inventoried by concept mapping [7] and added to the previous described three levels of evaluation criteria, reflecting a hierarchical complexity [Doesburg et al. [7] level 1–3, this study level 4] (Table 1).

### General design

The effect of contextual sensitivity in the visual evaluation of crystallization images was tested by comparing subjects’ performance while primed according to the two perceptional strategies (levels: analytical vs. kinesthetic engagement). Hereto, the subjects were assigned randomly to either group A or B. Subjects of group A were instructed to evaluate the crystallization images according to an analytical perceptual strategy (single object recognition) by adhering to the use of ‘atomic features’ (i.e. utilizing solely the criteria of the levels 1 and 2 in Table 1). Whereas subjects of group B were instructed to evaluate the images according to a kinesthetic engagement in Gestalt perception and a subsequent secondary, confirmatory ‘atomic feature’ evaluation (i.e. mainly levels 3 and 4 in Table 1). On day two the perceptual strategy was switched between the groups.

### Ranking decomposition levels of wheat

**Fig 1:**
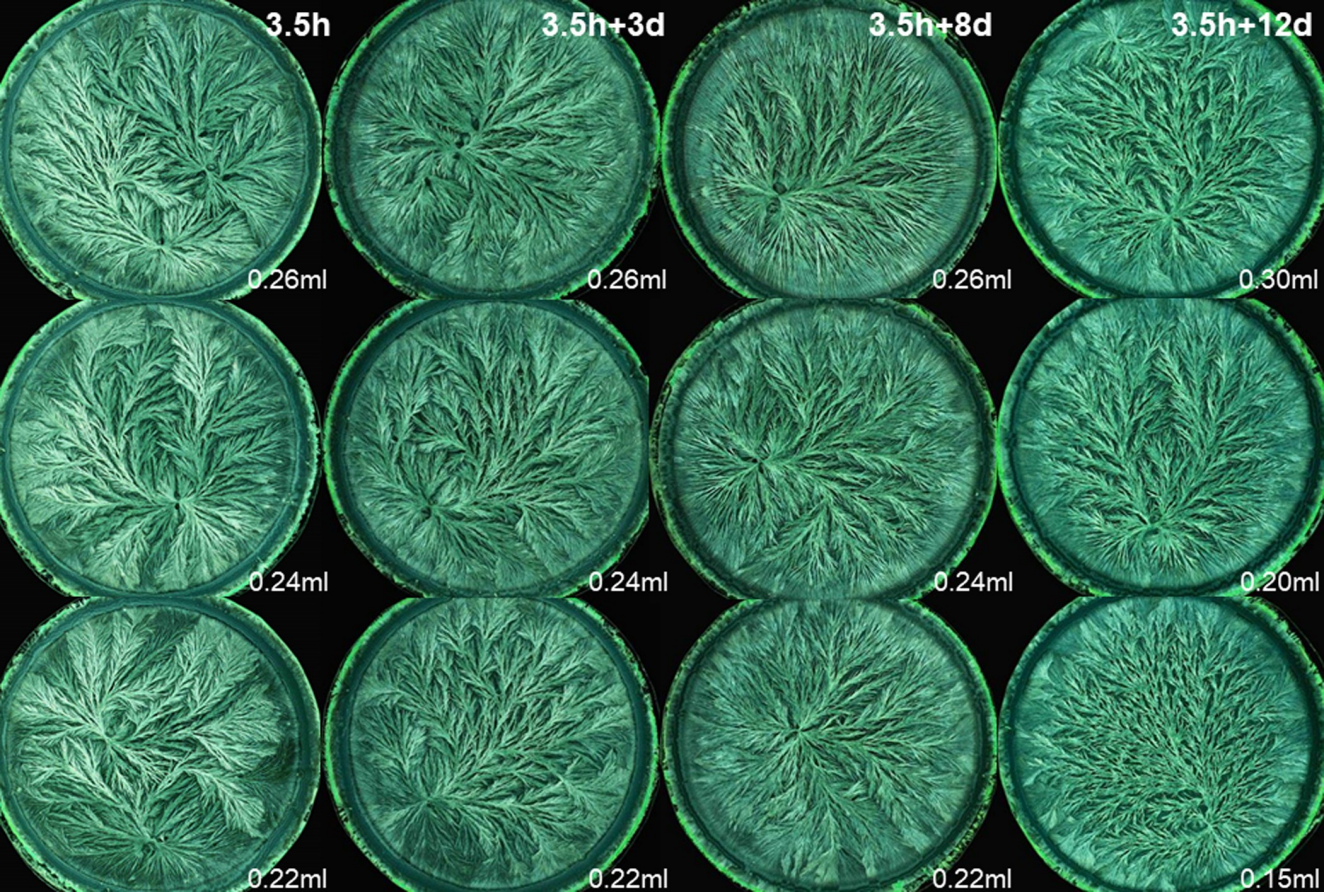
Wheat crystallization images for the Gestalt decomposition. Columns from left to right denote the four decomposition stages, ordered according to an increased intensity of controlled decomposition (“fresh”, 3, 8, and 12 days of controlled decomposition induced by storing the wheat extract at 8°C). The rows denote the three different wheat additive amounts tested (bottom row 0.22 ml, middle 0.24 ml, top 0.26 ml wheat extract per 160 mg CuCl_2_.2H_2_O per plate). The presented images originated from samples collected from the biodynamic production system.

**Fig 2:**
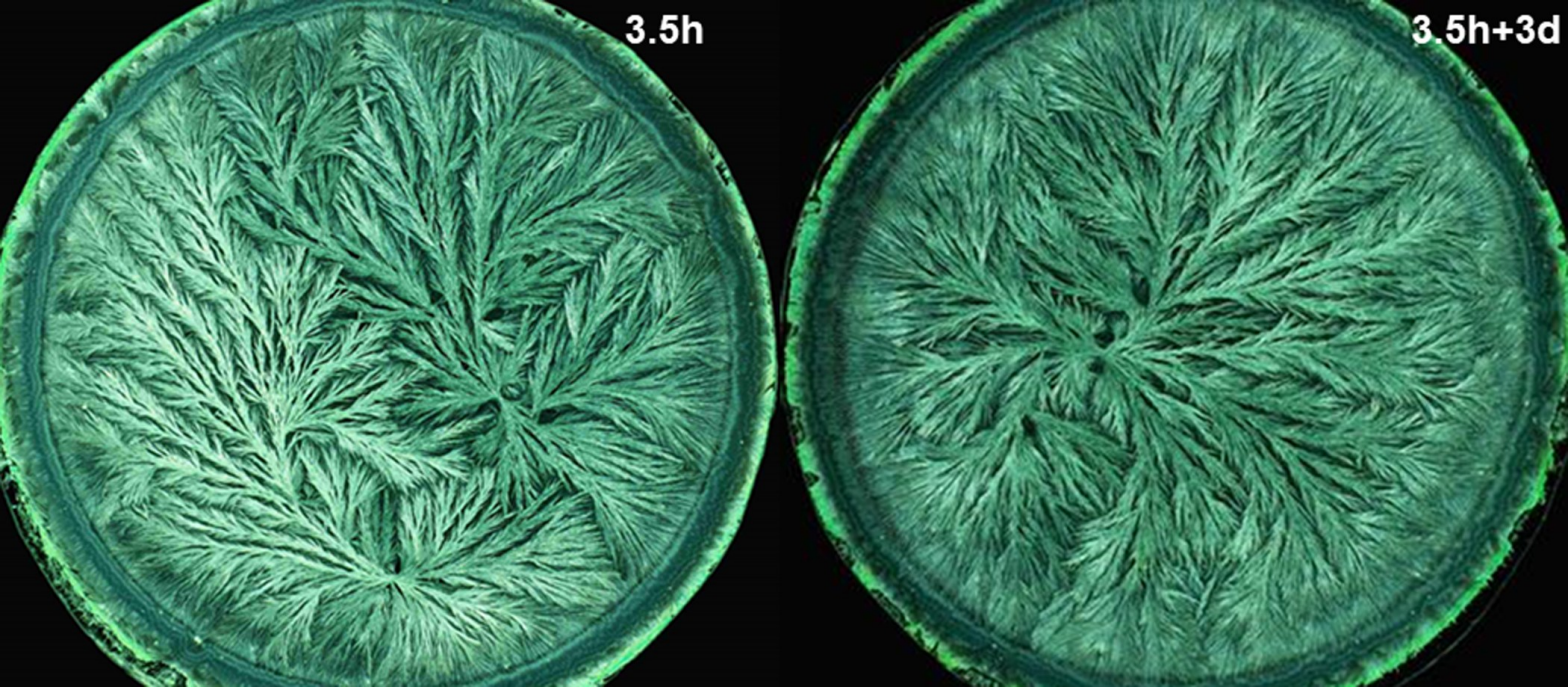
Enlargement of two crystallization images from Fig 1 to illustrate the criteria of Table 1. Left: “fresh” wheat extract, right 3d aged wheat extract. Mixing ratio 0.26 ml wheat extract per 160 mg CuCl_2_.2H_2_O per plate.

For the wheat exam, the panel was presented crystallization images representing four decomposition levels. In the examples in Figs 1 and 2, decomposition was perceivable on all four levels of visual evaluation criteria as depicted in Table 1. On the two levels of single morphological features, decomposition resulted in a decrease of the number of side needles, as indicated by an increase of the area that was not covered with crystal needles. Additionally, the side needles and branches exhibited a larger ramification angle. The crystallization images of the 12 days decomposed wheat extract failed to form clear branches at all. On the gesture level (level 3), a decrease was observed in the coordination ability from the crystallization center, in combination with a decrease in the perradiation and the organic curvature. The needle branches became less apparent, and were less able to reach the periphery of the dish. On the level of kinesthetic criteria, a loss of tension in the needle branches running from the center to the periphery and a decrease of a consistent dynamic in the filling of the plate was notable.

**Fig 3:**
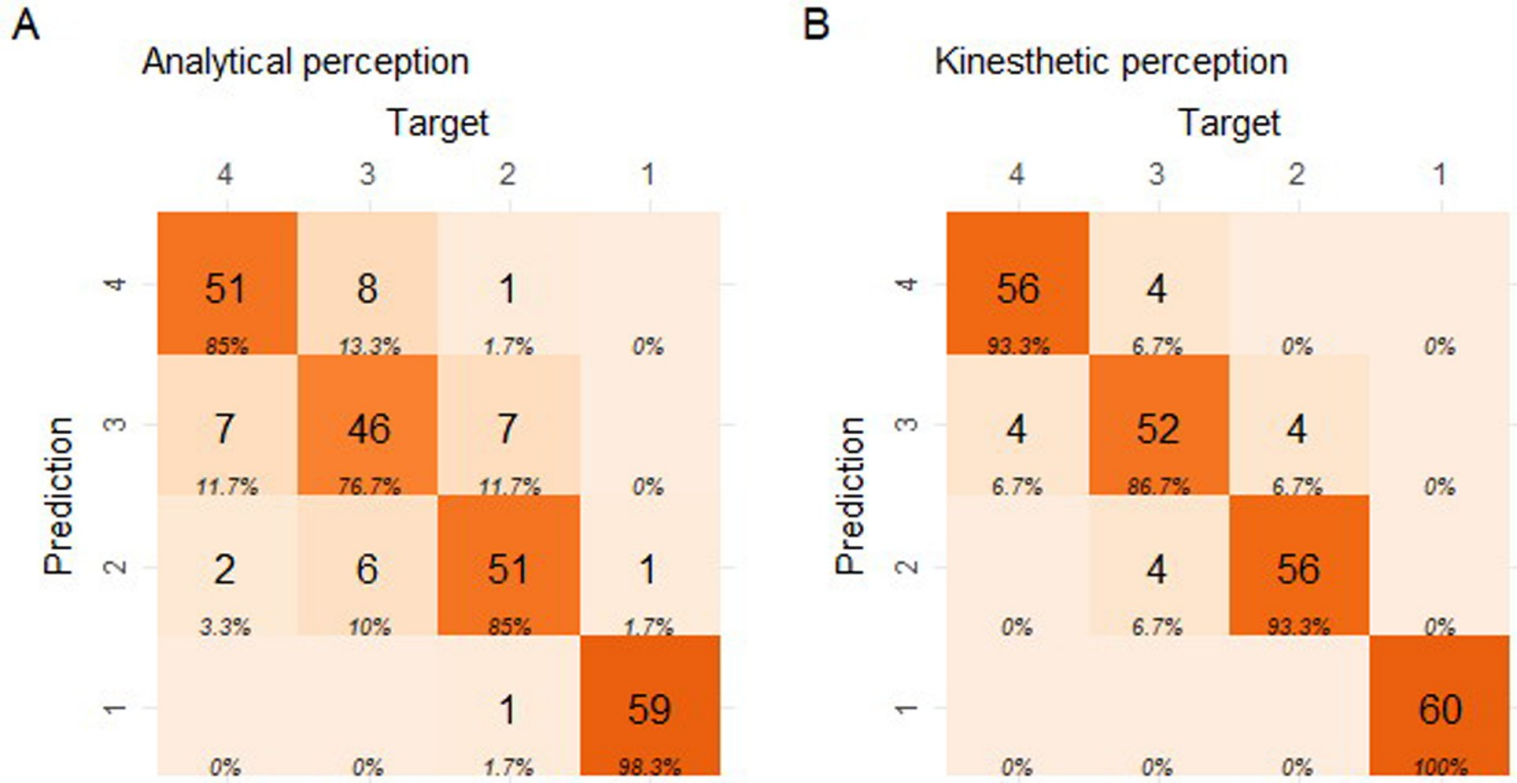
Confusion matrices for the wheat ranking test, representing the performance of the panels’ summed classifications according to the perceptual priming. Left: analytical priming, right: kinesthetic priming. The classes 1–4 indicate the decomposition levels (from low 1, to harsh 4) for both the prediction and the target (true class) values. Each tile contains the number of observations and the column percentage. The diagonal indicates the correct classification counts. Color intensity is based on the counts.

A first impression of the effect of the perceptual priming is obtained by plotting the summed classifications of the panel in confusion matrices (Fig 3).

The overall degree of correct classification was somewhat higher with a kinesthetic priming of the panel. Of specific interest was the complete lack of ‘2 class violations’ when the panel was kinesthetically primed.

**Fig 4:**
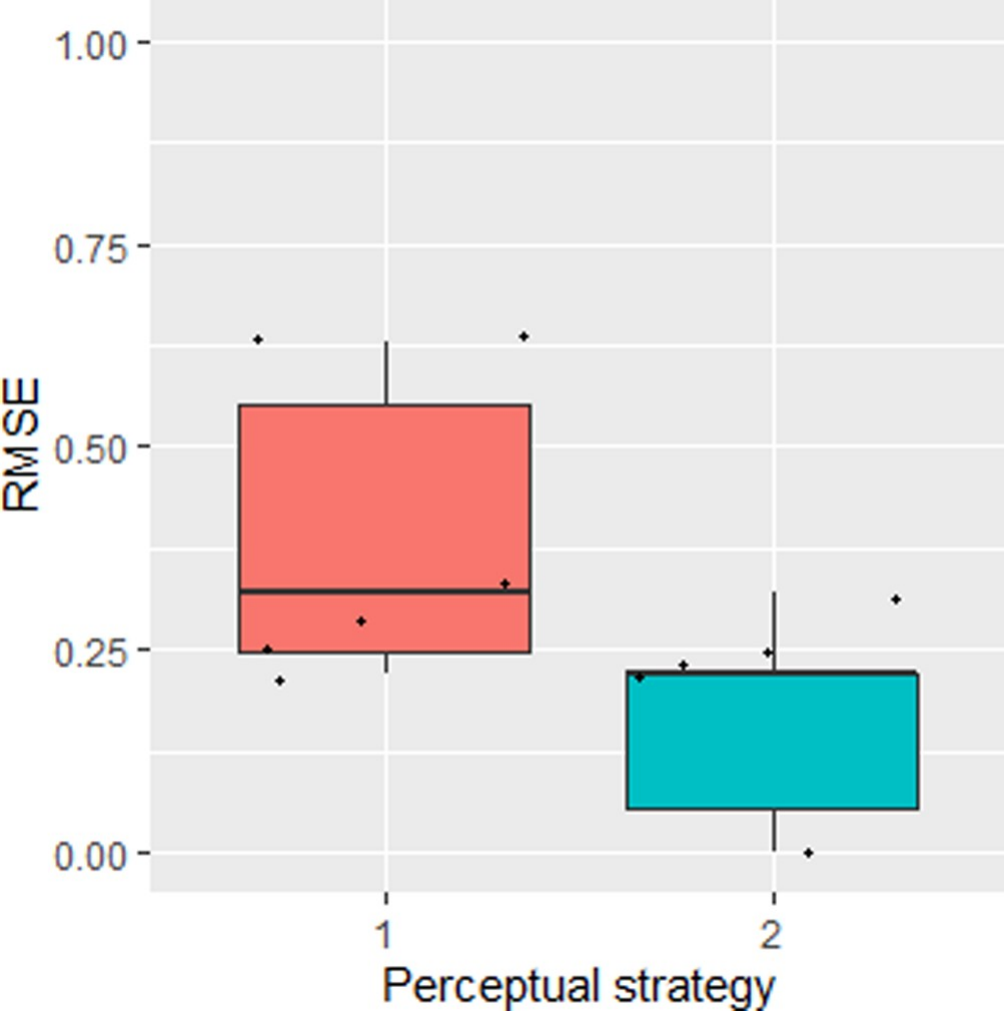
Root Mean Square Error (RMSE) as a function of the factor perceptual strategy (1 = analytical, 2 = kinesthetic engagement) in the performance of the classification of the wheat crystallization pictures. Jitter elements depict individual subjects’ RMSE values.

An overview of the population statistics is presented in Table 2. A graphical presentation of the RMSE as a function of the perceptual strategy (1 = analytical, 2 = kinesthetic engagement) in the performance of the classification, is depicted in Fig 4.

**Table 2:** Primed perceptual strategy (1 = analytical, 2 = kinesthetic engagement), number of subjects (n), mean and SD of the RMSE (Root Mean Square Error) calculated on the basis of subjects’ confusion matrices for the wheat ranking test.

Perceptual strategy	N	RMSE	
		Mean	SD
1	6	0.390	0.191
2	6	0.163	0.132

The mean RMSEs for the two perceptual strategies were compared with a paired sample t-test. The p-value of the t-test was 0.038, indicating that the perceptual priming of the panel had a significant effect on the mean RMSE. Additionally, the lower RMSE score for the kinesthetic engagement was indicative of a lower classification error.

### Ranking decomposition levels of rocket lettuce

**Fig 5:**
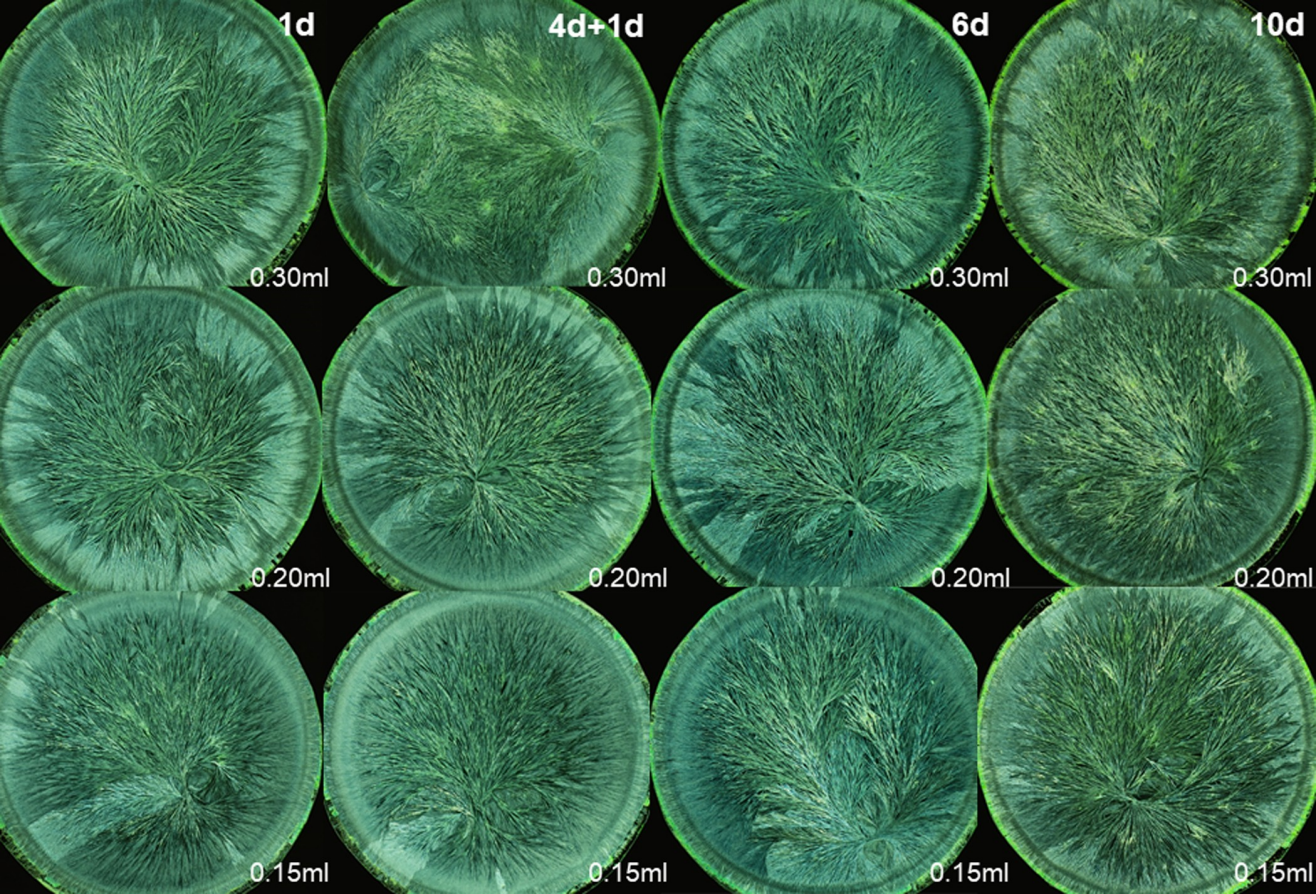
Rocket lettuce crystallization images for the Gestalt decomposition. Columns from left to right denote the four decomposition stages, ordered according to an increased intensity of controlled decomposition, induced by storing the rocket lettuce juices at 4°C: 1 day of juice aging, 4 days of leaf aging + 1 day of juice aging, 6 days of juice aging, and 10 days of juice aging. The rows denote the three different rocket lettuce additive amounts tested (bottom row 0.15 ml, middle 0.20 ml, top 0.30 ml rocket lettuce juice per 160 mg CuCl_2_.2H_2_O per plate). The presented images are all obtained from the ConMin production system with horn silica application [37].

**Fig 6:**
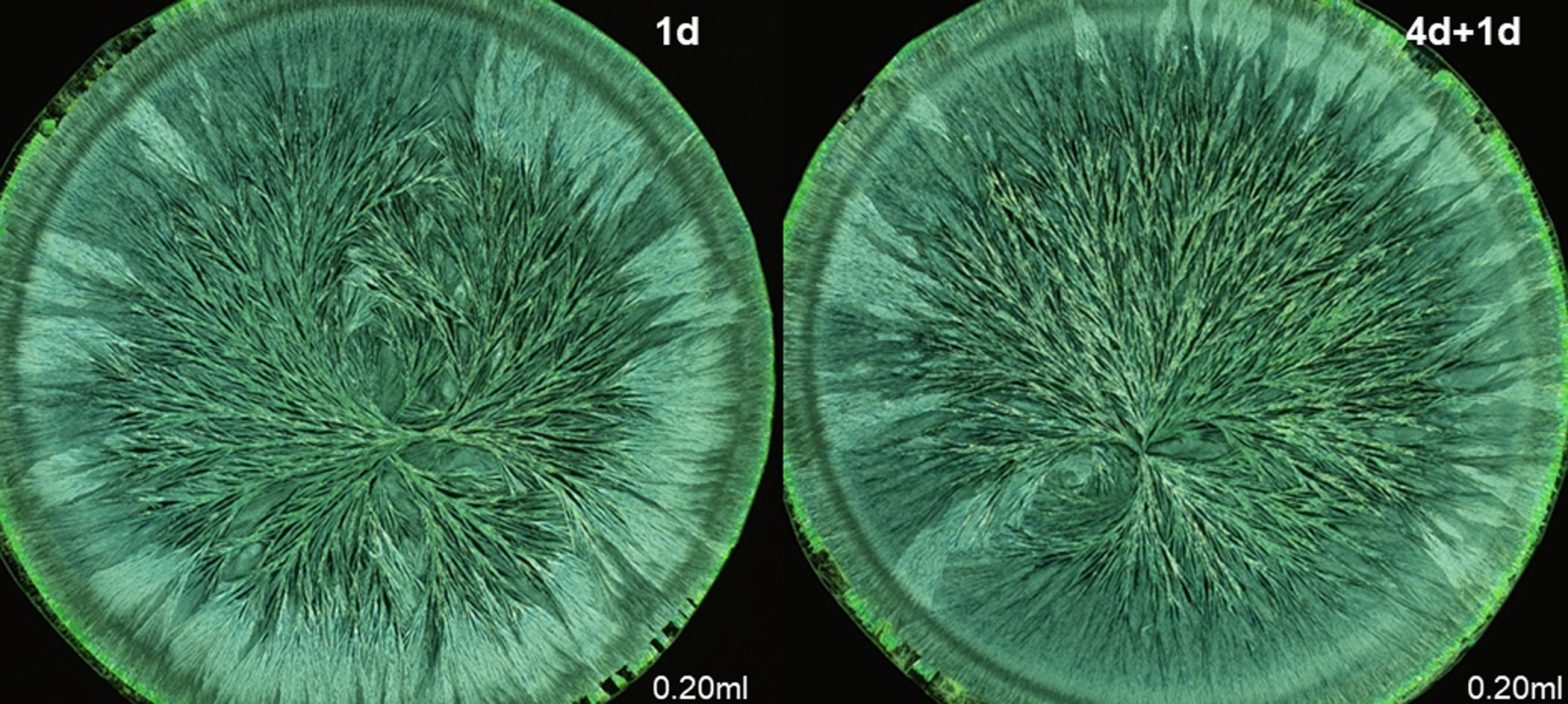
Enlargement of two crystallization pictures of Fig 5 to illustrate the criteria of Table 1. Left: 1 day aged rocket lettuce juice, right 4 days of leaf aging + 1 day of juice aging. Mixing ratio 0.20 ml rocket lettuce juice per 160 mg CuCl_2_.2H_2_O per plate.

The rocket lettuce exam in March 2018 contained four stages of decomposition. In the examples in Figs 5 and 6, differences on the two levels of single morphological features were smaller than for wheat.

The main feature that became apparent in response to decomposition was the appearance of so called ‘wickerwork’, i.e. parts of the crystal images exhibiting areas with fuzzily arranged, intertwined needles. Changes on the gestural and kinesthetic level were however more pronounced. A consistent decrease in motility, tension of the needle branches from the center to the periphery, fluent interconnected movement, and a sense of “presence” was notable in response to increased decomposition.

**Fig 7:**
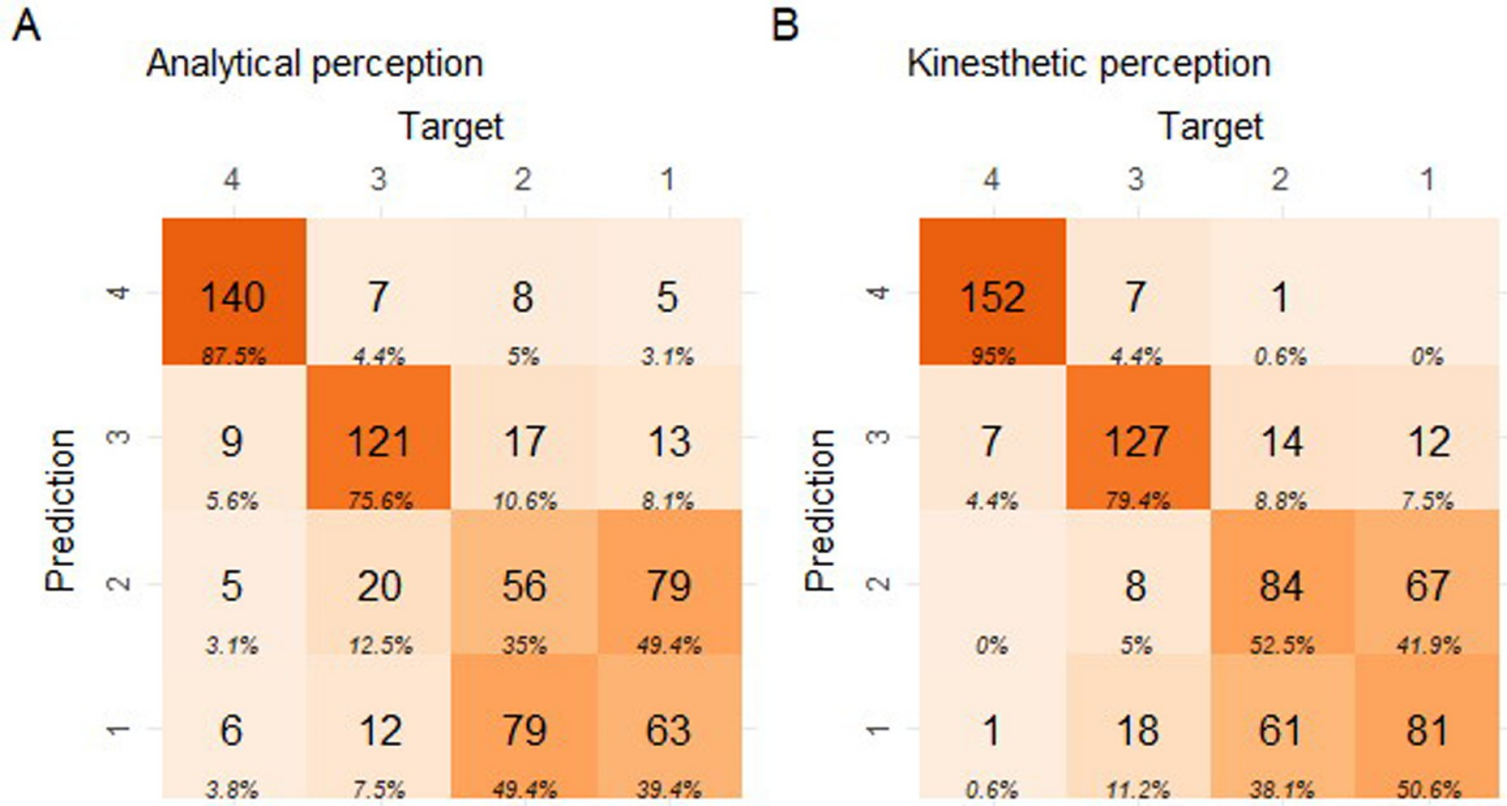
Confusion matrices representing the performance of the panels’ summed classifications according to the perceptual priming for the first rocket lettuce ranking test. Left: analytical priming, right: kinesthetic priming. The classes 1–4 indicate the decomposition levels (from low 1, to harsh 4) for both the prediction and the target (true class). Each tile contains the number of observations and the column percentage. The diagonal indicates correct classifications. Color intensity is based on the counts.

A first impression of the effect of the perceptual priming was obtained by plotting the summed classifications of the panel in confusion matrices (Fig 7).

**Fig 8:**
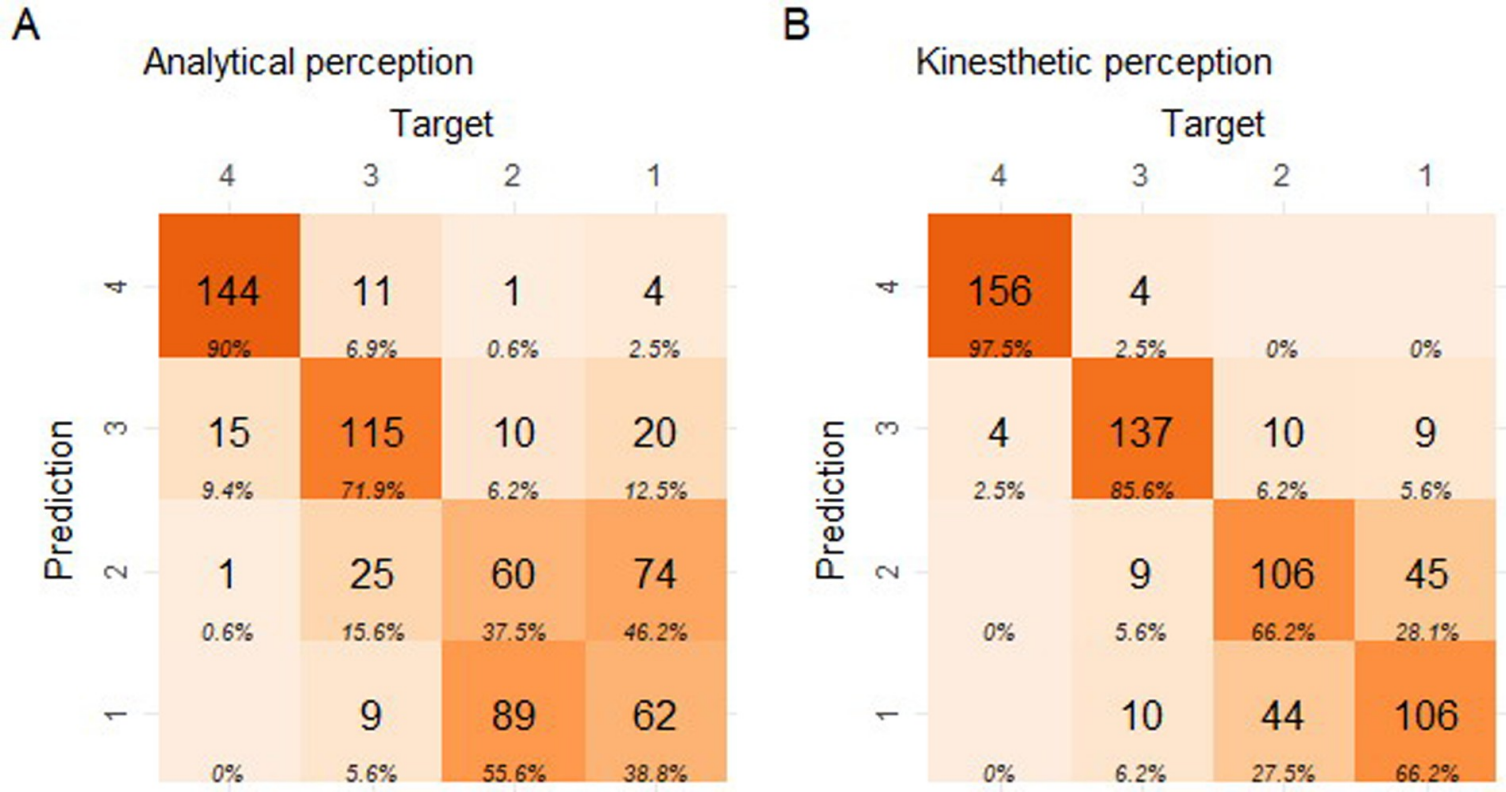
Confusion matrices representing the performance of the panels’ summed classifications according to the perceptual priming for the second rocket lettuce ranking test. Left: analytical priming, right: kinesthetic priming. The classes 1–4 indicate the decomposition levels (from low 1, to harsh 4) for both the prediction and the target (true class). Each tile contains the number of observations and the column percentage. The diagonal indicates correct classifications. Color intensity is based on the counts.

The overall degree of correct classification seemed marginally increased in response to a kinesthetic priming of the panel. This stimulated the panel to continue training. After an additional training period consisting of 6 x 1.5h online training sessions deploying supervised classification and peer tutoring by the best performing evaluators, the rocket lettuce panel test was repeated with the same exam set (exam 2, dd. September 27 and 28 2018) (Fig 8).

The overall degree of correct classification increaseed in response to kinesthetic priming of the panel. This was most pronounced in classes 1 and 2 (i.e. the two mildest decomposition levels).

**Fig 9:**
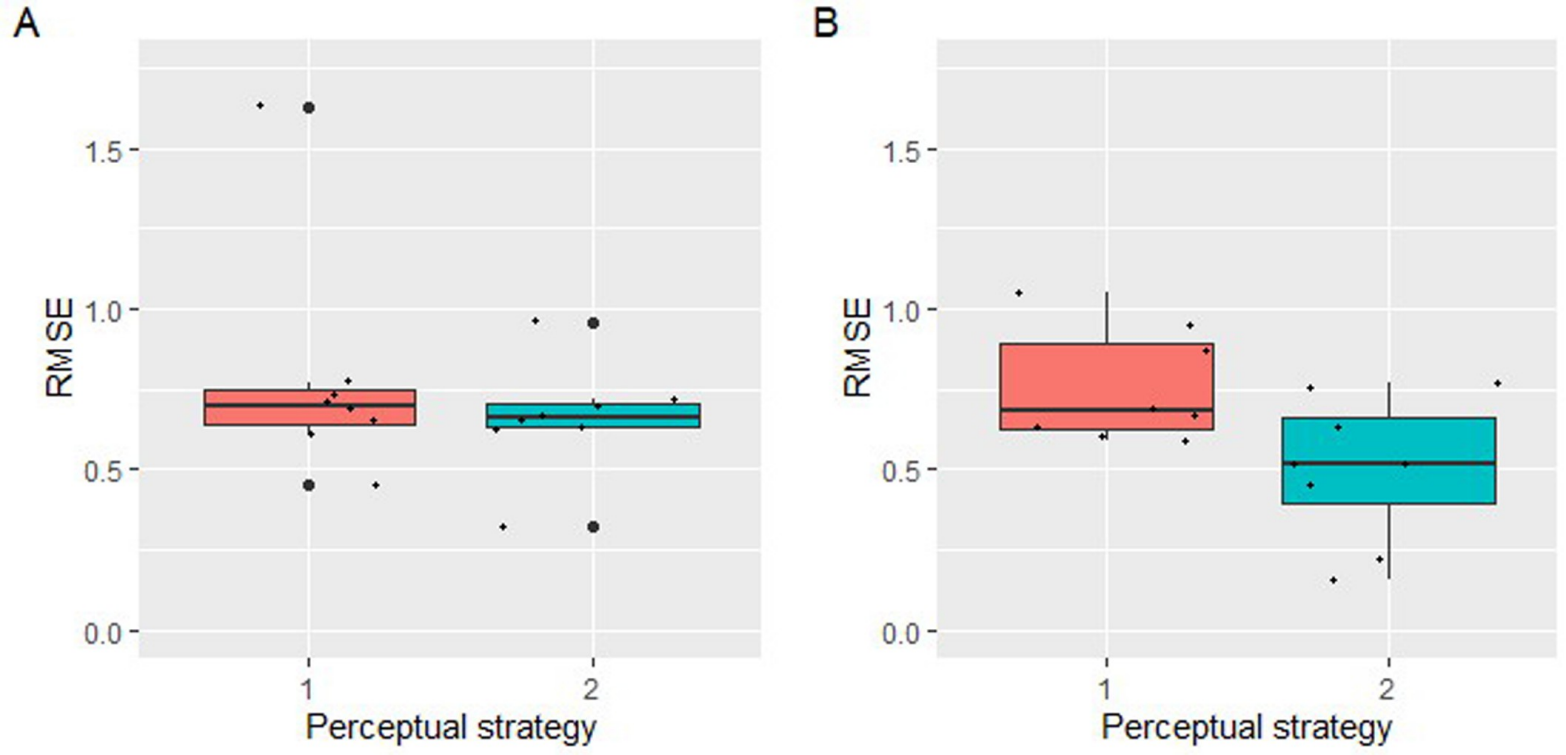
Root Mean Square Error (RMSE) as a function of the perceptual strategy (1 = analytical, 2 = kinesthetic engagement) in the performance of the classification of the rocket lettuce crystallization pictures. A: first exam (dd. March 13 and 14 2018); B second exam (dd. September 27 and 28 2018). Jitter elements depict individual subjects’ RMSE values.

An overview of the population statistics for both exam dates is presented in Table 3. A graphical presentation of the RMSE as a function of the perceptual strategy (1 = analytical, 2 = kinesthetic engagement) in the performance of the classification, is depicted in Fig 9A and 9B.

**Table 3:** Primed perceptual strategy (1 = analytical, 2 = kinesthetic engagement), number of subjects (n), mean and SD of the RMSE (Root Mean Square Error) calculated on the basis of subjects’ confusion matrices for the two successive rocket lettuce ranking tests.

	Perceptual strategy	N	RMSE	
			Mean	SD
Exam March 13 and 14 2018				
	1	8	0.781	0.357
	2	8	0.660	0.174
Exam September 27 and 28 2018				
	1	8	0.757	0.175
	2	8	0.504	0.225

The mean RMSEs for the two perceptual strategies were compared with a paired sample t-test. The p-value of the first exam date was 0.1824, indicating that the perceptual priming of the panel had no significant effect on the mean RMSE. In the second ranking test the p-value decreased to a border line significance of 0.055, indicating that perceptual priming did in fact have an effect on the mean RMSE. The lower RMSE score for the kinesthetic engagement indicated a lower classification error when the subjects were primed to rank according to a kinesthetic perceptual strategy.

## Discussion

### Perception in evaluation and accuracy of the results

In this article we researched the role of contextual sensitivity in the ranking of crystallization images of two agricultural products according to their degree of induced degradation. To this end, subjects were primed according to two perceptional strategies (levels: analytical vs. kinesthetic engagement), according to a within-subject design. The ranking tests consisted out of wheat and rocket lettuce crystallization images, exhibiting four levels of induced degradation.

The Root Mean Square Error (RMSE) was used as primary outcome criterion to assess the classification error. RMSE was calculated on the base of the subjects’ confusion matrices, thereby taking the absolute differences between actual and predicted class numbers into consideration.

Perceptual priming resulted in a significant difference in the mean RMSE for the wheat ranking test, (paired t-test p = 0.038), and in a borderline significant effect for the second rocket lettuce ranking test (p = 0.055). Kinesthetic priming consistently resulted in a lower mean RMSE score, indicating a lower classification error when the subjects classified the crystallization images according to this perceptual strategy.

While in the wheat crystallization images, pronounced differences were visible on all four hierarchical levels of visual evaluation criteria (Table 1); in rocket lettuce images, there were hardly any differences on the level of analytical criteria. This was mainly observed for the two mildest decomposition levels. Consequently, the largest difference in classification between the two perceptual strategies, was observed in class 1 and 2 of the confusion matrices (Fig 8). An explanation may be that decomposition processes in whole leaves proceed slower than in pressed juice, since the cell structure is still intact. Following this line of thought, this may indicate that small differences in decomposition are reflected more pronounced on the level of gestural and kinesthetic Gestalt criteria than on the level of single morphological criteria. Evidently, the verification of this assumption requires additional research.

Apparently, the perceptional strategy influences the accuracy of the results. The wheat performance test and the second rocket lettuce performance tests support the hypothesis that the application of a global perception, imbued with kinesthetic engagement, increases the ranking accuracy of decomposed samples. Despite the increased differentiation ability of the panel as a whole after adapting towards a kinesthetic Gestalt perception, the accuracy between the panel members still varied somewhat, as reflected in the jitter in the boxplots (Figs 4 and 9). It seems plausible that this is linked to each panel member’s level of expertise and personal preference [45–47].

### Kinesthetic engagement and the mirror neuron system

Kinesthetic engagement is essentially an association of an observer’s own kinesthetic sensation, with observed movements [16]. Kinesthetic engagement can be experienced by looking at people, living beings and even non-living objects. Kinesthetic engagement in crystallization image evaluation involves an embodied simulation of the growth, curvature and tension of the tree-like branches of the crystallization images [17]. While the embodied simulation is supposed to be the psychological mechanism responsible for kinesthetic engagement, the mirror neurons system is supposed to form the neural basis [15, 54]. Of major significance is that even the observation of static images of actions evokes a motor simulation of the gesture that is required to produce it [15, 16].

### Subjective perception and intersubjective assessment by panel training

In a field of research such as sensory analysis, it is necessary to take the individual subjective perception as the basis for the assessment. Subjective perception becomes inter-subjective through training and standardization of a panel and the applied descriptors [38]. Meanwhile this field of research has become a respected field of science with verifiable phenomena in sensory perception. In recent years sensory perception has further evolved to the inclusion of food-induced emotions [55, 56].

The ISO standards of sensory analysis were used for the methodical development of the visual evaluation of copper chloride crystallization images and the training of the panel (see methods chapter). By means of concept mapping [57] the individual subjective perceptions were rendered inter-subjective [7].

The Gestalt decomposition, appears as a salient unity of multiple emergent interrelations between local criteria and global features. The applied image analysis algorithms in computer evaluation rely either on the analysis of these local criteria [31], or on the spatial gray-level co-occurrence properties, like the degree of smoothness, coarseness, depth, and regularity of the crystallization texture [30]. It seems plausible that the reason for the superiority of the human visual evaluation in differentiating samples based on their resistance to decomposition lies in the inclusion of these above mentioned emergent interrelations in this type of evaluation.

## Conclusions

The results presented here support the hypothesis that evaluation with a kinesthetic engagement in Gestalt perception leads to a higher accuracy in the evaluation of decomposition levels in crystallization images than an analytical perception. To the best of our knowledge, this is the first report on the training and application of such a perceptual strategy in a visual evaluation panel.

## References

[1] Capuano E, Boerrigter-Eenling R, van der Veer G, van Ruth SM. Analytical authentication of organic products: an overview of markers. J Sci Food Agric. 2013;93(1):12–28. 10.1002/jsfa.5914 23070660

[2] European Commission. Council Regulation (EC) No 834/2007 of 28 June 2007 on organic production and labelling of organic products and repealing Regulation (EEC) No 2092/91. Official Journal of the European Union. 2007;189(1):1–23.

[3] Kahl J, Bodroza-Solarov M, Busscher N, Hajslova J, Kneifel W, Kokornaczyk MO, et al. Status quo and future research challenges on organic food quality determination with focus on laboratory methods. J Sci Food Agric. 2014;94(13):2595–9. 10.1002/jsfa.6553 24374910

[4] Aguilera JM. The food matrix: implications in processing, nutrition and health. Crit Rev Food Sci Nutr. 2019;59(22):3612–29. 10.1080/10408398.2018.1502743 30040431

[5] Jacobs DR, Tapsell LC. Food, not nutrients, is the fundamental unit in nutrition. Nutrition reviews. 2007;65(10):439–50-–50. 10.1111/j.1753-4887.2007.tb00269.x 17972438

[6] McClements DJ. Understanding and controlling the microstructure of complex foods: Elsevier; 2007.

[7] Doesburg P, Huber M, Andersen J-O, Athmann M, van der Bie G, Fritz J, et al. Standardization and performance of a visual Gestalt evaluation of biocrystallization patterns reflecting ripening and decomposition processes in food samples. Biological Agriculture & Horticulture. 2014;31(2):128–45.

[8] Fritz J, Athmann M, Andersen J-O, Doesburg P, Geier U, Mergardt G. Advanced panel training on visual Gestalt evaluation of biocrystallization images: ranking wheat samples from different extract decomposition stages and different production systems. Biological Agriculture & Horticulture. 2018;35(1):21–32.

[9] Fritz J, Athmann M, Kautz T, Köpke U. Grouping and classification of wheat from organic and conventional production systems by combining three image forming methods. Biological Agriculture & Horticulture. 2011;27(3–4):320–36.

[10] Fritz J, Athmann M, Meissner G, Kauer R, Geier U, Bornhütter R, et al. Quality assessment of grape juice from integrated, organic and biodynamic viticulture using image forming methods. OENO One. 2020;54(2):373–91.

[11] Fritz J, Athmann M, Meissner G, Kauer R, Köpke U. Quality characterisation via image forming methods differentiates grape juice produced from integrated, organic or biodynamic vineyards in the first year after conversion. Biological Agriculture & Horticulture. 2017;33(3):195–213.

[12] Kahl J, Baars T, Bugel S, Busscher N, Huber M, Kusche D, et al. Organic food quality: a framework for concept, definition and evaluation from the European perspective. J Sci Food Agric. 2012;92(14):2760–5. 10.1002/jsfa.5640 22407871

[13] Busscher N, Kahl J, Andersen J-O, Huber M, Mergardt G, Doesburg P, et al. Standardization of the Biocrystallization Method for Carrot Samples. Biological Agriculture & Horticulture. 2010;27(1):1–23.

[14] Huber M, Andersen J-O, Kahl J, Busscher N, Doesburg P, Mergardt G, et al. Standardization and Validation of the Visual Evaluation of Biocrystallizations. Biological Agriculture & Horticulture. 2010;27(1):25–40.

[15] Freedberg D, Gallese V. Motion, emotion and empathy in esthetic experience. Trends Cogn Sci. 2007;11(5):197–203. 10.1016/j.tics.2007.02.003 17347026

[16] Miyoshi K. What allows us to kinesthetically empathize with motions of non-anthropomorphic objects? The Journal of Somaesthetics. 2019;4(2).

[17] Waldburger B. Morphologie und Empathie- Studien zur Auswertung von Kristallisationsbildern. Elemente der Naturwissenschaft. 2009;90:80–91.

[18] Busscher N, Doesburg P, Mergardt G, Sokol A, Kahl J, Ploeger A. Influence of dewetting on the crystallization behavior of CuCl(2) in the presence of BSA during evaporation in a Petri dish. Heliyon. 2019;5(1):e01102. 10.1016/j.heliyon.2018.e01102 30627687 PMC6321889

[19] Busscher N, Doesburg P, Mergardt G, Sokol A, Kahl J, Ploeger A. Crystallization patterns of an aqueous dihydrate cupric chloride solution in the presence of different amounts of Bovine Serum Albumin. Journal of Crystal Growth. 2020;529:125272-.

[20] Busscher N, Kahl J, Ploeger A. From needles to pattern in food quality determination. J Sci Food Agric. 2014;94(13):2578–81. 10.1002/jsfa.6498 24281794

[21] Kahl J, Busscher N, Ploeger A. Questions on the Validation of Holistic Methods of Testing Organic Food Quality. Biological Agriculture & Horticulture. 2010;27(1):81–94.

[22] Kahl J, Andersen J-O, Athmann M, Busscher N, Doesburg P, Fritz J, et al. Laboratory intercomparison for biocrystallization (crystallization with additives) applied to different wheat varieties. Elemente der Naturwissenschaft. 2015.

[23] Kahl J, Busscher N, Doesburg P, Mergardt G, Will F, Schulzova V, et al. Application of Crystallization with Additives to Cloudy and Clear Apple Juice. Food Analytical Methods. 2016;10(1):247–55.

[24] Kahl J, Busscher N, Hoffmann W, Mergardt G, Clawin-Raedecker I, Kiesner C, et al. Development and Performance of Crystallization with Additives Applied on Different Milk Samples. Food Analytical Methods. 2013;7(7):1373–80.

[25] Kahl J, Busscher N, Mergardt G, Andersen J-O, Doesburg P, Arlai A, et al. Standardization and Performance Test of Crystallization with Additives Applied to Wheat Samples. Food Analytical Methods. 2015;8(10):2533–40.

[26] Szulc M, Kahl J, Busscher N, Mergardt G, Doesburg P, Ploeger A. Discrimination between organically and conventionally grown winter wheat farm pair samples using the copper chloride crystallisation method in combination with computerised image analysis. Computers and Electronics in Agriculture. 2010;74(2):218–22.

[27] Andersen JO, Huber M, Kahl J, Busscher N, Meier-Ploeger A. A concentration matrix procedure for determining optimal combinations of concentrations in biocrystallization. Elemente der Naturwissenschaft. 2003;79:97-.

[28] Andersen JO, Laursen J, Koelster P. A Refined Biocrystallization Method applied in a Pictomorphological Investigation of a Polymer. Elemente der Naturwissenschaft. 1998;68:1–20.

[29] Busscher N, Kahl J, Doesburg P, Mergardt G, Ploeger A. Evaporation influences on the crystallization of an aqueous dihydrate cupric chloride solution with additives. J Colloid Interface Sci. 2010;344(2):556–62. 10.1016/j.jcis.2009.12.045 20116068

[30] Andersen JO, Henriksen CB, Laursen J, Nielsen AA. Computerised image analysis of biocrystallograms originating from agricultural products. Computers and Electronics in Agriculture. 1999;22(1):51–69.

[31] Doesburg P, Nierop AFM. Development of a structure analysis algorithm on structures from CuCl2⋅2H2O crystallization with agricultural products. Computers and Electronics in Agriculture. 2013;90(0):63–7.

[32] Kahl J, Busscher N, Mergardt G, Mader P, Torp T, Ploeger A. Differentiation of organic and non-organic winter wheat cultivars from a controlled field trial by crystallization patterns. J Sci Food Agric. 2015;95(1):53–8. 10.1002/jsfa.6818 25044434

[33] Meelursarn A. Statistical evaluation of texture analysis from the biocrystallization method: Effect of image parameters to differentiate samples from different farming systems. PhD thesis, Witzenhausen: University of Kassel; 2007.

[34] Gallinet JP, Gauthier-Manuel B. Wetting of a glass surface by protein adsorption induces the crystallization of an aqueous cupric chloride solution. Journal of Colloid and Interface Science. 1992;148(1):155–9.

[35] Beckmann H. Über Keimbildung, Einkristallwachstum und Auffächerungswachstum von CuCl2*2H2O in rein-wässerigen und Eiweiß-haltigen Lösungen. PhD thesis, Rheinischen Friedrich Wilhelms Universität Bonn, 1959.

[36] Leray JL. Growth kinetics of hydrated cupric chloride. Journal of Crystal Growth. 1968;3:344–9.

[37] Athmann M. Produktqualität von Salatrauke (Eruca sativa L.) und Weizen (Triticum aestivum L.): Einfluss von Einstrahlungsintensität, Stickstoffangebot, Düngungsart und Hornkieselapplikation auf Wachstum und Differenzierung: Institut für Organischen Landbau, University Bonn; 2011.

[38] ISO 11035. Sensory analysis—Identification and selection of descriptors for establishing a sensory profile by a multidimensional approach. 1994.

[39] Collins English Dictionary. Definition of gestalt from the Collins English Dictionary [online]. New York: HarperCollins; 2020 [Available from: https://www.collinsdictionary.com/dictionary/english/gestalt.

[40] Wertheimer M. Laws of organization in perceptual forms: London: Routledge & Kegan Paul; 1923.

[41] Drew T, Evans K, Vo ML, Jacobson FL, Wolfe JM. Informatics in radiology: what can you see in a single glance and how might this guide visual search in medical images? Radiographics. 2013;33(1):263–74. 10.1148/rg.331125023 23104971 PMC3545617

[42] Krupinski EA. Visual scanning patterns of radiologists searching mammograms. Acad Radiol. 1996;3(2):137–44. 10.1016/s1076-6332(05)80381-2 8796654

[43] Wolfe JM, Vo ML, Evans KK, Greene MR. Visual search in scenes involves selective and nonselective pathways. Trends Cogn Sci. 2011;15(2):77–84. 10.1016/j.tics.2010.12.001 21227734 PMC3035167

[44] Van Belle G, De Graef P, Verfaillie K, Busigny T, Rossion B. Whole not hole: expert face recognition requires holistic perception. Neuropsychologia. 2010;48(9):2620–9. 10.1016/j.neuropsychologia.2010.04.034 20457169

[45] Cornejol C, Simonetti F, Aldunate N, Ibanez A, Lopez V, Melloni L. Electrophysiological evidence of different interpretative strategies in irony comprehension. J Psycholinguist Res. 2007;36(6):411–30. 10.1007/s10936-007-9052-0 17364233

[46] de Oliveira S, Nisbett RE. Culture Changes How We Think About Thinking: From "Human Inference" to "Geography of Thought". Perspect Psychol Sci. 2017;12(5):782–90. 10.1177/1745691617702718 28972847

[47] Ryu D, Abernethy B, Mann DL, Poolton JM. The contributions of central and peripheral vision to expertise in basketball: How blur helps to provide a clearer picture. J Exp Psychol Hum Percept Perform. 2015;41(1):167–85. 10.1037/a0038306 25485663

[48] Ashby FG, Maddox WT. Human category learning. Annu Rev Psychol. 2005;56:149–78. 10.1146/annurev.psych.56.091103.070217 15709932

[49] Galotti KM. Cognitive psychology in and out of the laboratory: SAGE publications Inc.; 2013. 10.1002/cncr.27902

[50] ISO 8589. Sensory analysis-General guidance for the design of test rooms. 2007.

[51] Cardoso JS, Sousa R. Measuring the performance of ordinal classification. International Journal of Pattern Recognition and Artificial Intelligence. 2011;25(08):1173–95.

[52] Core Team R. R: A language and environment for statistical computing. R Foundation for Statistical Computing. Vienna, Austria: URL https://www R-project org/[Google Scholar]. 2017.

[53] H W. ggplot2: Elegant graphics for data analysis. Springer-Verlag, New York, USA, pp. 260. 2016.

[54] Keysers C, Wicker B, Gazzola V, Anton JL, Fogassi L, Gallese V. A touching sight: SII/PV activation during the observation and experience of touch. Neuron. 2004;42(2):335–46. 10.1016/s0896-6273(04)00156-4 15091347

[55] Geier U, Bussing A, Kruse P, Greiner R, Buchecker K. Development and Application of a Test for Food-Induced Emotions. PLoS One. 2016;11(11):e0165991. 10.1371/journal.pone.0165991 27861503 PMC5115674

[56] Spinelli S, Masi C, Dinnella C, Zoboli GP, Monteleone E. How does it make you feel? A new approach to measuring emotions in food product experience. Food Quality and Preference. 2014;37:109–22.

[57] Swanborn PG. Evalueren: Boom Koninklijke Uitgevers; 2004.

